# Cellulose-Based Composite Materials for Fresh Water Extraction from Atmospheric Air

**DOI:** 10.3390/polym17030328

**Published:** 2025-01-25

**Authors:** Dmitry Repin, Mariia Gablina, Natalya Repina, Kirill Cherednichenko, Wenpeng Li, Yuliiya Gushchina, Evgenii Ivanov, Vyacheslav Melnikov, Rawil Fakhrullin, Vladimir Vinokurov

**Affiliations:** 1 Department of Physical and Colloidal Chemistry, National University of Oil and Gas «Gubkin University», Leninsky Prospekt 65, Moscow 119991, Russiavladimir@vinokurov.me (V.V.); 2 Faculty of Chemistry, Lomonosov Moscow State University, Leninskie Gory 1, Moscow 119991, Russia; 3 Institute of Fundamental Medicine and Biology, Kazan Federal University, Kreml uramı 18, Kazan 420008, Republic of Tatarstan, Russia; 4 Institute for Regenerative Medicine, Sechenov First Moscow State Medical University (Sechenov University), Moscow 119991, Russia

**Keywords:** atmospheric water harvesting, BMIMCl, hybrid material, ionic liquid, microfibrillated cellulose, MFC, polystyrene, PS

## Abstract

The fibrous hybrid material was synthesized by suspension radical styrene polymerization on the surface of cellulose microfibers. The resulting material was used to prepare a thermally stable and mechanically strong porous composite matrix that was employed as a carrier for further precipitation of the hygroscopic agents: CaCl_2_ and 1-butyl-3-methylimidazolium chloride. The obtained composite materials were used to capture atmospheric water at different relative humidity levels and extract fresh water. A composite material containing an ionic liquid (1-butyl-3-methylimidazolium chloride) as a hygroscopic agent demonstrated the best water absorption efficiency and reusability potential.

## 1. Introduction

Continuous supply of fresh water is a critical requirement for the sustainable development of modern civilization. Fresh water accounts for only 2.5% of the total 1.4 × 10^18^ tons of water on the planet, while the majority of this water is frozen in glaciers, ice caps, and permafrost [1]. The natural sources of fresh water are presented by lakes, rivers, and soil waters. Nevertheless, due to their uneven geographical distribution and the ever-growing demand on fresh water, the elaboration of alternative methods of fresh-water extraction has become an issue during the last decades. The purification/recycling of wastewater is a widely applied alternative approach to retrieve fresh water. Unfortunately, a number of the technological solutions related to water purification (mechanical, chemical, biological, and physicochemical methods) require large capital investment and high material and energy consumption, and often do not meet high environmental standards [2].

Extraction of fresh water from atmospheric water vapor is a promising way to supply fresh water since it does not strongly depend on the geography of the region. Devices for the collection of water from air by sorption are generally categorized into two systems: passive and active [3,4,5]. Passive systems rely on natural energy sources and do not require an additional power supply. In these systems, the cycle lasts 24 h: moisture is absorbed at night, and during the day, solar heat drives desorption, followed by distillation. Active systems, in contrast, can perform multiple absorption and desorption cycles within a single day, thereby increasing water production. However, this increased efficiency comes at the cost of higher energy consumption due to the use of electricity [6]. During recent decades, atmospheric water harvesting (AWH) materials, including hygroscopic inorganic compounds, functional hydrogels, ionic liquids (ILs), and metal–organic frameworks (MOFs) have attracted increasing attention [1,7,8]. Despite their excellent water absorbency, each of the above classes of AWH materials has application limitations and none can be considered ideal.

Thus, for example, hygroscopic salts have a strong dipole–dipole force that results in a great water adsorption capacity (5–6 g·g^−1^) [9]. G.E. William et al. applied CaCl_2_, which has the capacity to absorb up to 97% of its weight in moisture, as a desiccant by employing a solution of salt [10]. Hygroscopic salts absorb moisture through hydration; however, they are prone to agglomeration and the formation of inactive layers on the surface of particles [11]. This can reduce permeability to moisture and, consequently, decrease the amount of water absorbed. Another issue is the liquefication of hygroscopic salts upon moisture absorption leading to equipment corrosion and environmental concerns [11,12]. Despite zeolites and silica gel being first-generation water sorption materials, they are characterized by high regeneration temperatures (above 100 °C) and low moisture absorption rates. Unlike zeolites, silica gel and hygroscopic salts of monovalent/divalent metals, MOFs can capture more water from the ambient air with a relative humidity of only 20% and do not require high temperatures for moisture desorption. A number of effective MOF-based AWH systems have been proposed recently [13,14,15,16]. However, their high cost, poor resistance to water and toxicity, which can present a danger to human health, are the bottlenecks restricting their AWH application [17,18]. Polymer hydrogels possess an excellent water absorption and can take any shape, but they have low capacity, poor physical strength, and may be subject to chemical leaching [19]. ILs are viscous fluids composed of anions and cations. They possess high hygroscopicity due to their ionic nature. The ions in the liquid create a strong electrostatic field that attracts polar water molecules. Besides electrostatic interaction, many ILs are capable of forming hydrogen bonds with water molecules, thus, enhancing water sorption [20]. However, ILs have several disadvantages, including strict requirements for their storage and transportation conditions (e.g., precise temperature and humidity control) to prevent decomposition or alteration of their properties [21]. Rather high cost of ILs can also considerably impede their practical application [20]. The leakage of IL is the last but not the least issue of this class of AWH materials.

Various approaches have been elaborated on to overcome these limitations of AWH materials [22]. For example, the employment of various supports/carriers of AWH materials can considerably enhance the atmospheric water absorption and exclude their leakage. For instance, a hydrogel layer was deposited onto an MOF/solar absorber layer to suppress convective heat loss [23]. In another research study, impregnation of the porous supports by hydroscopic salts or ILs helped to avoid their leakage [20]. Moreover, deposition of rather dense IL on the support with a high specific surface significantly enhanced the moisture absorption of this AWH material. Integration of hydroscopic CaCl_2_ into an alginate-derived matrix enhanced AWH performance and provided cyclic stability of the obtained composite [24]. However, the current ecological concerns place strict demands on the environmental impact of moisture absorbents and supports for AWH materials, which has pushed researchers to use various eco-friendly/biodegradable materials [25].

Cellulose is a biopolymer that has been gaining interest worldwide for its biodegradability, non-toxicity, water affinity and large surface area [26]. Cellulose is one of the most abundant biopolymers, which is the reason for its low price. Owing to the high inter- and intramolecular hydrogen bonds and van der Waals forces, this biopolymer possesses high hydrophilicity and mechanical stability, which makes it an ideal component for AWH materials [25]. For instance, cellulose and its derivatives (in particular nanocellulose) have been frequently employed to prepare various hydrogels for agricultural uses (collection and sustained release of water), collection of oil spills, and drug delivery [25,27,28,29]. To enhance water absorption, nanocellulose-based hydrogels were modified with hydroscopic salts [30,31]. Indeed, cellulose is not the only biopolymer that can be used to create hydroscopic materials and carriers for AWH materials. Chitosan (made by treating the chitin shells of shrimp and other crustaceans with alkaline substances) can also be employed to synthesize water-absorbing hydrogels. For instance, chitosan-based hydrogels demonstrated effective water adsorption and considerable enhancement of structural durability upon repeated use [32]. Molybdenum carbide/carbon-based chitosan hydrogel (MoCC-CH) was recently designed for effectively absorbing atmospheric water and also efficiently accelerating water evaporation by tuning the biopolymer content in the hydrogel [33]. However, despite the high water sorption capacity of biopolymer-based hydrogels, their synthesis often involves expensive raw materials (e.g., nanocellulose), complex procedures, and large-scale challenges [34].

However, the wide application of nanocellulose is currently limited by its sophisticated and expensive production [35]. In this regard, employment of cheaper cellulose microfibrils can be a possible solution to apply biopolymer-based materials in industry. To the best of our knowledge, there are no reports on the application of the cellulose microfibrils as AWH material or as possible porous supports for commercial hydroscopic agents. Unlike nanocellulose, distinguished by shorter and more rigid structures, the fibrillar form exhibits an enhanced ability to form three-dimensional networks due to the efficient interactions between fibrils [36]. This makes it more suitable for the development of reinforcing composites and biomaterials while maintaining lightness and porosity.

This work aims to describe the synthesis of the composite materials based on a porous hybrid microfibrillated cellulose/polystyrene (MFC/PS) matrix and moisture-absorbing agents: CaCl_2_ and 1-butyl-3-methylimidazolium chloride. Variation in biopolymer/polymer mass ratio allowed us to achieve optimal mechanical characteristics and shape the stability and hydrophilicity of the porous matrix. The obtained composites have been applied as new AWH materials for the first time. MFC/PS blends exhibited passive water vapor absorption from the atmosphere requiring minimal energy input. The MFC/PS-based composites represent a simple and cost-efficient alternative to nanocellulose-based AWH materials, while maintaining high water absorption and reliable performance across a wide range of humidity conditions.

## 2. Materials and Methods

### 2.1. Materials

Styrene (ρ = 0.906 g/cm^3^ at 20 °C, purity ≥ 99.0%, stabilized with 4-tert-butylpyrocatechol), ammonium persulfate ((NH_4_)_2_S_2_O_8_, purity ≥ 97%), acetone ((CH_3_)_2_O, purity ≥ 99.5%), 1-butyl-3-methylimidazolium chloride (BMIMCl, purity ≥ 95%) and calcium chloride (CaCl_2_, CC, purity ≥ 97%) were purchased from Sigma-Aldrich (St. Louis, MO, USA). Softwood pulp in the form of bleached sulfate pulp was supplied by Arkhangelsk PPM (Arkhangelsk, Russia).

Microfibrillar cellulose (MFC) was obtained from bleached paper pulp according to the procedure described previously [37]. Pulp water suspension (3 wt.%) of the paper pulp was treated with a supermasscolloider MKCA 6-5 grinder (Masuko, Sangyo Co., Ltd., Kawaguchi, Japan) in 7 working cycles. The resulting suspension in water was used without modification for the emulsion polymerization of styrene.

### 2.2. Synthesis of Hybrid Fibrils

**Figure 1:**
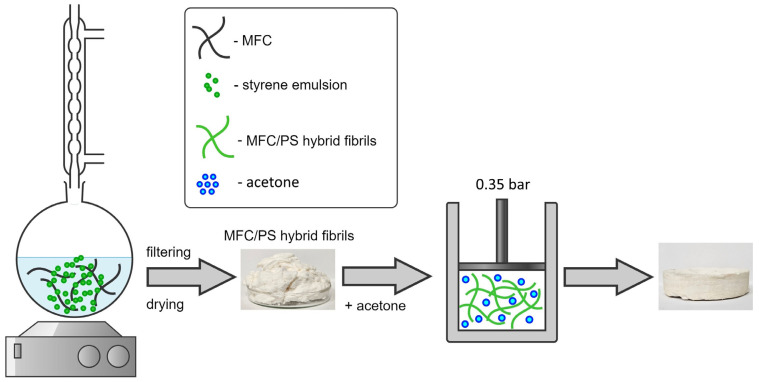
The scheme of synthesis of MFC/PS hybrid fibrils and composite porous matrix. The obtained MFC/PS hybrid fibrils during the first stage were compacted in a piston-cylinder with the addition of a small amount of acetone.

The deposition of polystyrene (PS) onto cellulose fibrils was provided in accordance with a procedure described previously [37,38]. Styrene was added to 3 wt.% MFC water suspension; the mass ratio of MFC:styrene was 70:30. The obtained solution was heated up to 60 °C and ammonium persulfate (0.5 wt.% with respect to styrene) was added to it at constant stirring. The synthesis lasted 240 min, the obtained white product was filtered, washed and freeze dried. The corresponding synthesis scheme is presented in Figure 1.

### 2.3. Preparation of Composite Materials

The porous composite matrices were prepared from 10 g of well-ground hybrid fibrils. MFC/PS was soaked with 15 mL of acetone (118 wt.% in relation to MFC), loaded in a piston-cylinder (diameter of 100 mm) and pressed up to 0.35 bar for 1 min using a universal testing machine TRM-O in manual mode (see Figure 1). No blowing agent was used. The retrieved samples were dried at 55 °C until a constant mass was reached.

Two composite materials were produced by impregnating a porous matrix with AWH materials presented by BMIMCl and CC. A piece of porous matrix (10 g) was placed in a 3M CC solution containing 10 g of CC for 24 h until the solution was completely absorbed. Subsequently, the resulting composite was dried at 60 °C until a constant weight was attained. The composite sample containing BMIMCl was obtained by deposition of 10 g of molten BMIMCl onto the composite matrix for 5 min. Then, the obtained composite sample was dried for 1 h in a forced air-drying cabinet (Binder, Camarillo, CA, USA) at a humidity level of 25%. For convenience, the samples of MFC/PS matrix modified with CC and BMIMCl will be denoted as MFC/PS/CC and MFC/PS/BMIMCl, correspondingly.

### 2.4. Methods

#### 2.4.1. Electron Microscopy

The morphology of the hybrid fibrous material as well as the porous matrix and composite materials were investigated using scanning electron microscopy (SEM) using a JIB 4501 multi-beam microscope (JEOL Ltd., Tokyo, Japan). The samples were fixed on the SEM stubs using double-sided carbon tape. The surface of the samples was coated with 20 nm Au layer using a Q150R ES Plus ion sputtering system (Quorum Technologies Ltd., Laughton, UK) to avoid undesired surface charging of the samples and the appearance of corresponding artifacts in SEM micrographs. SEM micrographs were obtained in the SE mode at an accelerating voltage of 5 kV in the magnification range of ×40–×1500.

#### 2.4.2. Fourier-Transform Infrared Spectroscopy (FTIR)

The FTIR measurements of the composite samples were performed using a Nicolet iS 10 FTIR spectrometer with germanium ATR crystal (Thermo Fisher Scientific, Waltham, MA, USA). All spectra were acquired in absorption mode in the 4000–600 cm^−1^ range. The spectral resolution was 8 cm^−1^, the acquisition time was 14 s. The OMNIC Thermo Scientific software (version 7.3) was employed to record and process the acquired data.

#### 2.4.3. The Porosity Study

The density, porosity, and water absorption coefficient values were evaluated for the pristine porous composite matrix and the matrices were modified with AWH agents using the liquid displacement method [37]. Distilled water was used as the displacement liquid. The sample was immersed in water for 5 min until it was saturated at a temperature of 26 °C and a humidity of 25%. The pore volume and porosity were measured three times, and the average value of the obtained indicators was taken as the test result.

The total pore volume (V_p_) and porosity (μ) were calculated using Formulas (1) and (2):

V_p_ = (M_wet_ − M_dry_)/ρ(H_2_O), (1)

µ = (V_p_/V) × 100%, (2)

where M_wet_ is the mass of the wet sample (g), M_dry_ is the mass of the sample before immersion (g), V is the volume of the sample before immersion (cm^3^), and ρ(H_2_O) is the density of distilled water (g/cm^3^) at the study temperature.

The water absorption coefficient (E) was calculated using Formula (3):

E = (M_wet,24_ − M_dry_)/M_dry_, (3)

where M_wet,24_ the mass of the wet sample after 24 h of composite soaking in distilled water (g).

#### 2.4.4. Study of Mechanical Characteristics

The tensile mechanical strengths of the pristine porous matrix and the obtained composite materials were studied using a TPM-O testing machine (Tochline, Moscow, Russia) at a speed of 50 mm/min, a temperature of 26 °C, and a humidity of 25%. The samples of 175 mm in length and 5 mm in thickness were prepared and tested.

The shape stability of the porous matrix is of utmost importance during multiple sorption/desorption cycles. To investigate it, the dimensions and weights of the three samples of dry pristine porous matrix (diameter = 100 mm, height = 100 mm, mass = 38.5 g) were noted. Then, the matrix samples were soaked in water for 24 h at 33 °C and dried in forced air-drying cabinet (Binder, USA) at a humidity level of 25% at three different temperatures: 27 °C, 55 °C, and 100 °C. The load of 16 kg was applied for 1 h to each sample to test porous matrix stability after the soaking/desorption cycle. When the load was removed, the corresponding parameters (dimensions and weight) of each sample were noted again.

#### 2.4.5. Thermogravimetric Analysis (TGA)

The thermal stabilities of the pristine MFC/PS porous matrix and the matrices impregnated with BMIMCl and CC were studied using TGA using a STA 449 F5 Jupiter simultaneous thermal analyzer (NETZSCH Instruments, Selb, Germany). The obtained data were compared with the corresponding TGA data acquired for the starting materials (MFC and PS). The experiment was carried out in a nitrogen atmosphere with a heating rate of 10 °C/min in 32–600 °C temperature range.

#### 2.4.6. Study of Water Absorption/Desorption

**Figure 2:**
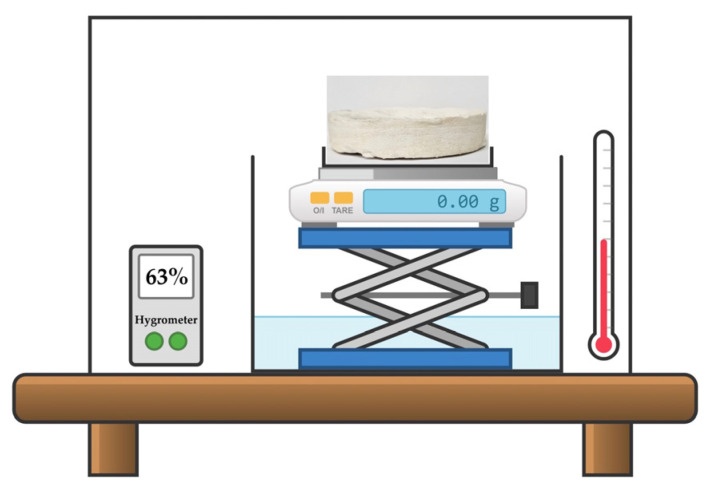
The scheme of water absorption experiment. The sample of pristine (or modified) MFC/PS matrix was placed under a transparent cover over a container of water at constant humidity (φ = 28%, 63%) and temperature. The weight of the sample was measured every 10 min.

The study of moisture sorption from the air was performed for the pristine composite porous matrix and matrices impregnated with AWH agents under a transparent cover over a container with water (250 mL) at room temperature. Figure 2 presents the scheme of the experiment. The mass ratio of the CC/BMIMCl:porous matrix was 50:50. All tested samples had a cylindrical shape (diameter = 100 mm) and mass of 20 g; however, the height of matrices impregnated with AWH agents was 26 mm, while the height of the pristine porous composite matrix was 52 mm. The experiment was conducted under conditions of low (φ = 28%) and high (φ = 63%) ambient humidity. The weight of sorbed water was registered every 10 min during the first 150 min of the experiment and in its end after 24 h.

The water desorption rates of the pristine composite porous matrix and matrix modified with AWH agents were determined in a forced air-drying cabinet (Binder, USA) at a humidity level of 8% and temperature of 55 °C. The experiment was terminated when the samples reached a constant mass.

## 3. Results and Discussion

### 3.1. Structure Investigation

**Figure 3:**
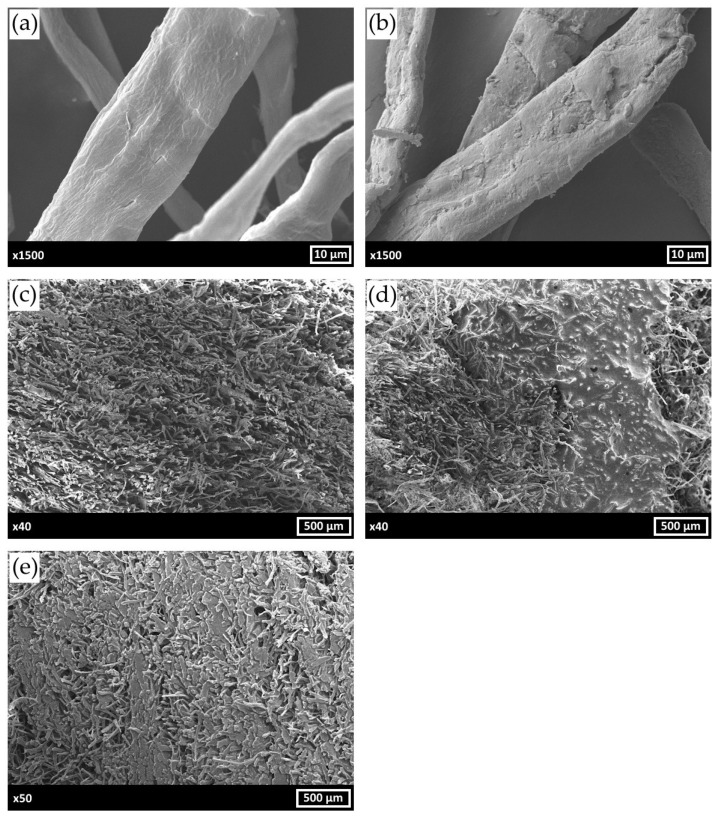
SEM micrographs of bare MFC (**a**), MFC/PS hybrid fibrils (**b**), MFC/PS pristine porous composite matrix (**c**), MFC/PS/CC (**d**), MFC/PS/BMIMCl (**e**).

The morphology and structure of the obtained hybrid fibrils, pristine composite porous matrix and the matrices impregnated with CC and BMIMCl were assayed using SEM (Figure 3). According to Figure 3a,b, PS was deposited as a uniform layer on the surface of cellulose fibrils. These results are in a good agreement with our previous observations [37].

Investigation of composite cross-sections revealed the different types of porous matrix impregnation by CC and BMIMCl (Figure 3c–e). CC forms a glaze-like layer on the porous matrix surface without anchoring within its pores. In contrast, BMIMCl exhibits uniform distribution throughout matrix voids, which accounts for the absence of agent-loss tendency during the multi-cycle operation. Such a distribution pattern of BMIMCl contributes to the material’s stability and retention of sorption properties over a repeated cycles of use.

**Figure 4:**
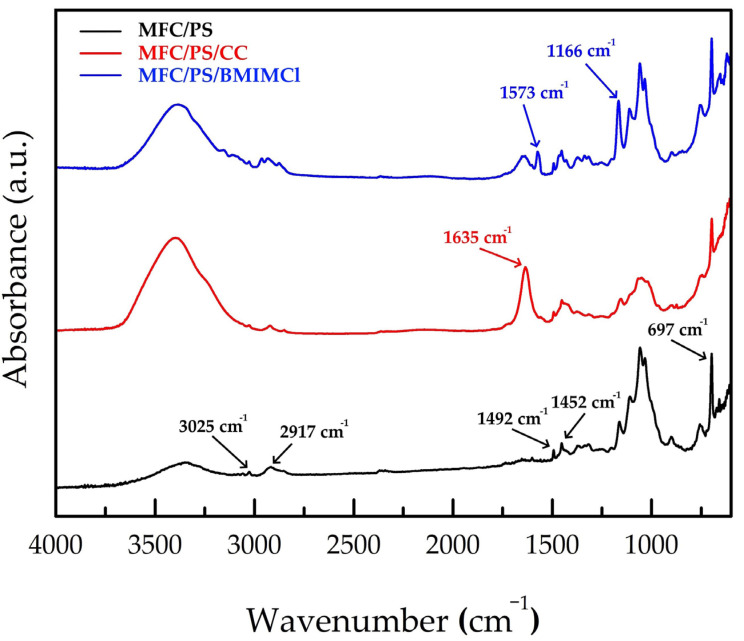
FTIR spectra of MFC/PS, MFC/PS/CC, and MFC/PS/BMIMCl. The wavenumbers of PS and BMIMCl characteristic bands are underwritten.

In accordance with our previous findings, no chemical interaction was observed between the deposited PS and cellulose microfibrils [37] (see Figure 4 and Table 1). The bands at 3025 cm^−1^, 2917 cm^−1^, 1492 cm^−1^, 1452 cm^−1^, and 697 cm^−1^ were attributed to PS [39], while bands in 3600–3200 cm^−1^ and 1400–900 cm^−1^ wavenumber ranges were attributed to glucose OH groups and glucose ring oscillations, correspondingly [37]. The impregnation of the MFC/PS matrix with CC did not bring any dramatic changes to the FTIR spectrum except from rising intensities of the bands at 3600–3300 cm^−1^ and at 1630 cm^−1^ referring to -OH stretching vibrations and H-O-H bending vibrations of crystal water in CC [40]. The new bands at 1573 cm^−1^ and 1166 cm^−1^ were observed in the MFC/PS/BMIMCl FTIR spectrum. According to the literature data, the observed bands were attributed to the imidazolidinium framework vibrations and C-N stretching vibrations, respectively [41]. As shown in Figure 4, no change in MFC and PS bands has been observed in FTIR spectra of MFC/PS/CC and MFC/PS/BMIMCl which supports assumption about the absence of any chemical interaction.

As previously demonstrated, PS amount significantly affects the density (ρ), pore volume (V_p_), porosity (µ), and water absorption coefficient (E) of the porous matrix [37]. Matrix impregnation with AWH materials can significantly change these characteristics. As shown in Table 2, the initial matrix is characterized by high porosity and low density. Nevertheless, one should note that these parameters can be considerably influenced by molding conditions. The presence of 70 wt.% hydrophilic MFC in the matrix increased the water absorption coefficient, which provides rapid diffusion of water into the volume of the material. Meanwhile, despite rather low PS content, the composite matrix has excellent shape stability after prolonged exposure to water, similar to the samples described earlier [37]. Such a high shape stability allows the development of new systems for moisture capture from air on the basis of the hybrid materials.

Modification of the matrix by AWH materials significantly affected the porosity and density values of the obtained composites. As shown in SEM images, CC glazes the pores of the matrix, resulting in a threefold decrease in total pore volume and porosity compared to the pristine MFC/PS. In contrast, BMIMCl addition led to porosity and pore volume increase. This phenomenon can be explained by the ability of BMIMCl to interact with cellulose fibrils, making matrix pore space more malleable and capacious. The density value of MFC/PS/BMIMCl composite is greater than the same values of the MFC/PS matrix and MFC/PS/CC by 8.8 and 1.8 times, respectively, which also demonstrates the better permeation of BMIMCl inside the porous matrix.

### 3.2. Thermal Stability Studies

**Figure 5:**
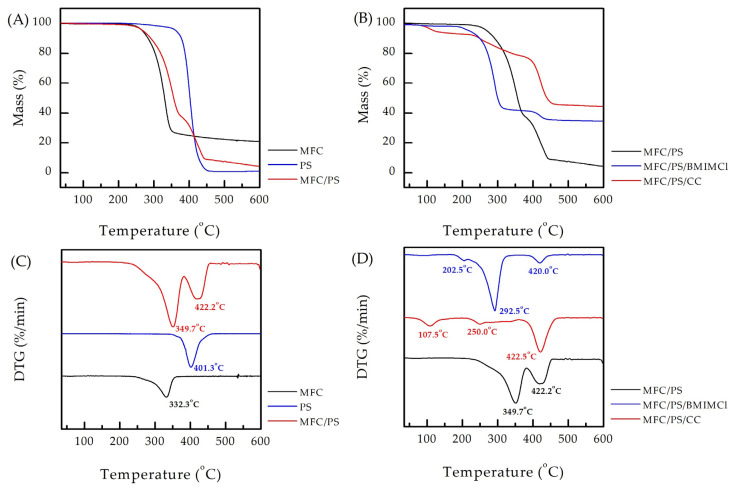
TGA and dTGA curves of (**A**,**C**) MFC, PS, MFC/PS, (**B**,**D**) MFC/PS, MFC/PS/CC, and MFC/PS/BMIMCl. The corresponding dTGA peaks are underwritten.

The TGA data were used to quantify the composition of the obtained composite porous matrix as performed before [37]. According to the obtained data, the content of MFC in the obtained matrix does not exceed 70 wt.%, which is in good agreement with the calculated value (see Figure 5).

The thermal stability of the obtained composite porous matrix is of great importance since the desorption procedure can include temperature rising to 90 °C. According to Figure 5A,C, the thermal decomposition of the matrix occurs as a result of successive processes of dehydration of bound moisture at 80–110 °C, followed by destruction of the cellulose component at 250–350 °C and PS decomposition at 350–450 °C. Interestingly, the both dTGA peaks of the components of the MFC/PS porous matrix are shifted towards higher temperatures compared to the data obtained for the bare materials (by 17.4 °C for MFC and by 20.9 °C for PS). The same phenomenon has been observed earlier [38] and can be explained by the different kinetics of the material thermal decomposition, which depends on its amount in the sample.

The TGA and corresponding dTGA curves of the MFC/PS matrix and matrices impregnated with AWH agents are presented in Figure 5B,C. In disagreement with the literature data [42], MFC/PS/CC loses water contained in CC in 50–200 °C. The decomposition of PS in MFC/PS/CC undergoes the same temperature as in the pristine matrix, whereas the decomposition of MFC is not pronounced. The dTGA curve of MFC/PS/BMIMCl contains three peaks referring to water evaporation (202.5 °C), cellulose thermal degradation (292.5 °C), and PS thermal degradation (420.0 °C). The cellulose decomposition at lower temperatures can be explained by the partial disruption of the cellulose crystalline regions by BMIMCl [43]. It was thus demonstrated that both impregnated matrices exhibited adequate thermal stability up to 200 °C, which is the most significant temperature range for potential future implementation.

### 3.3. Study of MFC/PS Matrix Mechanical Strength and Cyclic Stability

**Figure 6:**
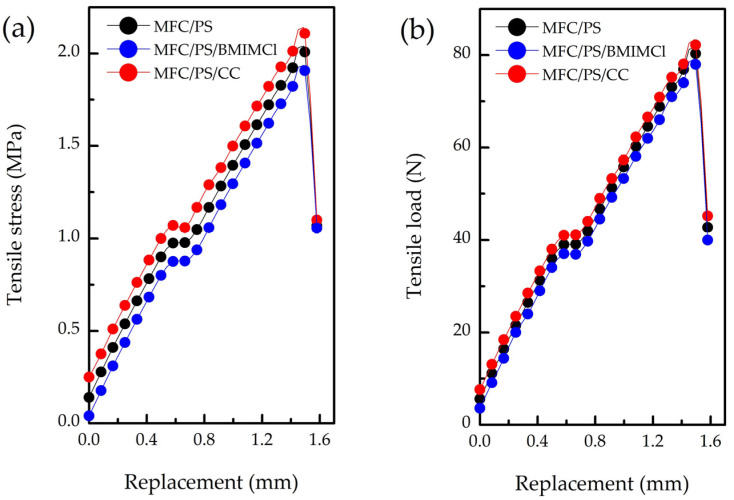
Compressive strength performance of pristine and modified MFC/PS matrices: (**a**) tensile strength, (**b**) tensile load.

The results of the mechanical strength properties investigation of the pristine composite matrix and modified matrix are presented in Table 3 and Figure 6.

Despite the fact that PS content in the pristine composite porous matrix is 30 wt.%, the mechanical properties of the matrix significantly differ from those of pure PS. For instance, the tensile strength (σ_max_) of pure PS is 10 times higher than the corresponding value of the obtained composite porous matrix, which is first of all linked with its high porosity.

According to Table 3, incorporation of CC in the porous composite matrix led to a slight enhancement of the strength characteristics of composite material compared to the pristine matrix. It can be explained by CC deposition on the surface of the matrix and its vitrification. Unlike CC, impregnation of the porous matrix by BMIMCl resulted in the tensile strength decline. This phenomenon can be attributed to the chemical interaction between BMIMCl and cellulose hydroxyl groups, explaining the better distribution of BMIMCl over the matrix volume. Meanwhile, the chemical binding of BMIMCl with hydroxyl groups can lead to cellulose fibrils softening [44] and, hence, partial disintegration of MFC in a composite matrix.

The integrity of the composite porous matrix is of paramount importance with regard to the stability of the final composite, particularly during its cyclic operation. It was found that the dimensions of the pristine matrix did not change after the load of 16 kg was applied to the matrix sample, after it was subjected to the cycle of water soaking/desorption.

**Figure 7:**
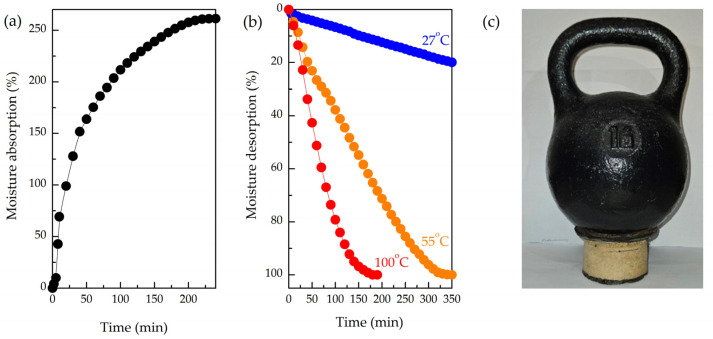
(**a**) Sorption of MFC/PS porous matrix immersed in water; (**b**) desorption at different temperatures; (**c**) demonstration of MFC/PS porous matrix shape stability after 10 sorption/desorption cycles under load of 16 kg.

According to Figure 7a, the maximum rate of water absorption is reached within the initial 30 min when the pristine porous composite matrix was immersed in water for 24 h. Then, the absorption rate gradually decreased, reaching a plateau after 3 h of observation. Furthermore, the composite matrix displayed outstanding shape stability even when subjected to prolonged exposure to water, thereby facilitating the potential use of this material in AWH systems for extended operational periods.

It is well known that various types of AWH agents require different regeneration temperatures that could reach rather high values. For instance, MOF-303 employed in [35] for vapor harvesting was regenerated at 55–60 °C. Thus, the stability of the MFC/PS matrix at different regenerating temperatures is of great practical importance. In this regard, the desorption of the MFC/PS matrix was carried out at 27 °C, 55 °C, and 100 °C.

As follows from Figure 7b, the MFC/PS matrix ability to desorb accumulated moisture strongly depends on the temperature. Thus, desorption occurs more rapidly at 100 °C, achieving complete desorption within 3 h, whereas desorption at 55 °C takes 6 h. The rate of moisture desorption at room temperature was extremely low (4–5 g/h). The initial mass of the MFC/PS matrix was restored only after 48 h. It should be noted that none of the applied temperature regimes had no effect on the composite matrix stability.

### 3.4. Moisture Absorption and Desorption Under the Influence of AWH Agents

The summary of moisture absorption tests under low and high humidity levels carried out for the pristine MFC/PS matrix, MFC/PS/BMIMCl, and MFC/PS/CC is presented in Table 4.

The MFC/PS matrix exhibited a modest absorption capacity under both low and high humidities, whereas the incorporation of CC and BMIMCl led to a substantial increase in atmospheric water capture.

**Figure 8:**
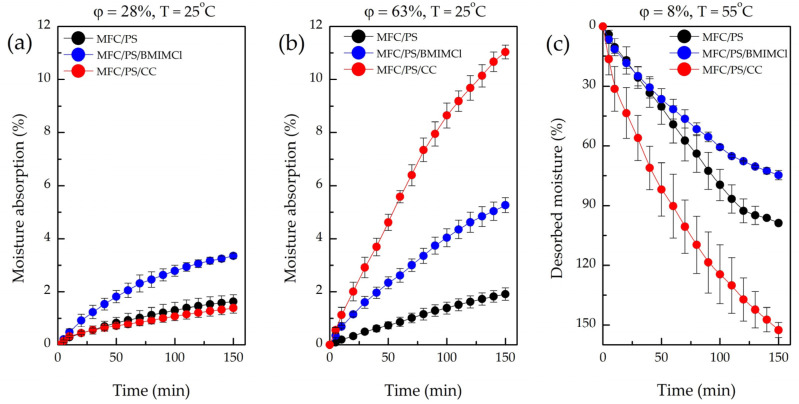
Moisture absorption from the air by pristine MFC/PS, MFC/PS/BMIMCl, and MFC/PS/CC at (**a**) low (28%) and (**b**) high (63%) humidity and 25 °C; (**c**) desorption curves of pristine MFC/PS, MFC/PS/BMIMCl, MFC/PS/CC at 55 °C and humidity of 8%.

In conditions of low humidity, the rate of moisture absorption of MFC/PS/BMIMCl is higher than that of MFC/PS/CC (Figure 8a). Interestingly, the weight of the adsorbed moisture by MFC/PS/CC is close to the same value of the pristine MFC/PS matrix even after 24 h.

In conditions of high humidity (Figure 8b), the sample with CC exhibited rapid moisture uptake within 150 min, while MFC/PS/BMIMCl absorbed only half of that water weight. During the next hours of observation, CC leakage was detected. However, after 24 h of the experiment, the absorbed water weights by both composites were found to be comparable (see Table 3).

The observed differences in the absorption curves of MFC/PS/BMIMCl and MFC/PS/CC can be explained by the various ways the AWH component is distributed within the porous matrix structure. CC (Figure 3d) demonstrates a tendency to glaze the surface of cellulose fibers without anchoring within the pores of the MFC/PS matrix. Since CC is situated closer to the surface of the composite, the rate of water vapor absorption is significantly greater than that of MFC/PS/BMIMCl. However, the water retained by CC does not penetrate inside the porous matrix, inducing salt liquefaction and subsequent leakage. Conversely, water vapor absorption by MFC/PS/BMIMCl is slower due to more uniform distribution of BMIMCl in the matrix volume (Figure 3e) owing to interaction with cellulose hydroxyl groups. Therefore, water absorbed by the MFC/PS/BMIMCl sample is homogeneously distributed throughout the composite volume and, thus, is better retained.

As shown in Table 4 and Figure 8c, the desorption behavior of the MFC/PS matrix and two composites within the first 150 min of the experiment was completely different. Thus, for instance, the sample containing CC lost 153% of its weight per absorbed water weight. Such an unusual mass reduction in the MFC/PS/CC sample can be explained by the loss/leakage of the part of the AWH agent during absorption, which is in agreement with the visual observations. MFC/PS/BMIMCl released only 75% of the captured water, which is less than the value of the pristine MFC/PS matrix. It should be underlined that even after 24 h of drying at 55 °C and a humidity of 8%, MFC/PS/BMIMCl still kept 5% of the captured water. In fact, the reason for this phenomenon is that BMIMCl forms both a strong electrostatic interaction and a hydrogen bonding with the adsorbed water molecules.

The MFC/PS/BMIMCl sample demonstrated outstanding moisture absorption ability compared to the pristine MFC/PS matrix. Unlike CC, no leakage of AWH agent was observed during the moisture absorption/desorption cycle.

A significant challenge of the modern AWH systems is posed by the fact that the desorption stage necessitates a considerable amount of energy input, given that the entire sorbent solution must be heated [26]. The heating of sorbents is typically accomplished through the use of electricity. However, some researchers have suggested the use of solar energy, taking into account its abundance and net zero carbon emissions [35,45]. It should be noted that obtained MFC/PS/BMIMCl composite can effectively desorb 95% of accumulated water at a relatively low temperature (55 °C), which can be reached using solar radiation. The possibility of the use of an alternative energy source as well as a high content of biodegradable cellulose in the obtained composite material makes it an attractive candidate for the modern AWH technologies complying with strict requirements for sustainable development of the economy.

The combination of the hydrophilic (MFC) and hydrophobic (PS) nature of the components of the obtained composite porous matrix improves both the water absorption and release functions. The non-polar PS chains repel the polar water molecules, while the polar MFC chains attract the water molecules [46]. The composite matrix, based on MFC and PS, overcomes the key limitations of existing AWH technologies, offering a sustainable, efficient, and cost-effective method for capturing atmospheric water, thus contributing to the fight against water scarcity.

## 4. Conclusions

The elaboration and wide application of environmentally friendly polymeric materials can reduce widespread ‘plasticization’ and pave the way for the development of ‘green technologies’ in this area of industrial production. Hybrid composite based on microfibrillar cellulose and polystyrene is an effective carrier for moisture capture systems from atmospheric air thanks to its high mechanical strength, porosity, and hydrophilicity. The moisture capture process is provided by using conventional hygroscopic materials (calcium chloride and ionic liquids) as the active components. The distribution of ionic liquid in the porous matrix structure significantly affects the porosity and mechanical properties of the composite material due to the better permeability of BMIMCl and partial dissolution of cellulose fibrils. The water absorbed by the sample with BMIMCl is homogeneously distributed throughout the volume of the composite, ensuring better retention in the pores. The composite material undergoes intensive desorption of water within less than 6 h at temperatures above 55 °C, allowing multiple cyclic uses for water absorption at night and extraction under sunlight during the day without additional energy inputs.

One of the key factors determining the performance characteristics of atmospheric water harvesting (AWH) technology is the absorption efficiency of composite material, as well as its ability to release accumulated moisture from the porous matrix. A promising approach to addressing these challenges involves doping a cellulose matrix with polystyrene and conductive materials, such as carbon felt, carbon nanotubes, melanin, or polyaniline. These materials are characterized by dark surfaces, which, theoretically, could enable the described material to enhance desorption efficiency by harnessing solar radiation.

The described composite matrix can be applied in portable water harvesting devices:

- Lightweight and energy-efficient systems for remote areas with limited access to water infrastructure, utilizing the composite’s passive absorption and solar-driven desorption properties;
- Building-Integrated Systems: Incorporating the composite into roofing or facade materials to harvest atmospheric water for domestic or irrigation use, particularly in arid or semi-arid regions;
- Emergency and Disaster Relief: Deployable units for clean water collection in disaster-stricken or drought-affected areas, where conventional water sources are unavailable;
- Agricultural use: Installation in greenhouses or open fields to collect water vapor for irrigation, reducing dependency on traditional water sources;
- Advanced Industrial Systems: Application in cooling systems or industrial processes requiring efficient humidity control, with potential for energy savings and water recovery.

Despite outstanding performance of the atmospheric water absorption by the obtained composites, the issues related to calcium chloride retention must be addressed. One of the possible resolutions is a doping of the composite matrix with additional sorbent or moisture-retaining substance (e.g., polyacrylamide).

## Figures and Tables

**Table 1:** The description of IR characteristic bands of cellulose, PS, CC and BMIMCl.

	Wavenumber, cm^−1^	Description	Ref.
MFC	1400–900	Glucose ring oscillations	[37]
	3700–3200	Glucose OH stretching oscillations	
PS	697	C-H out-of-plane bending oscillations	[39]
	756		
	1700–1400	Aromatic ring oscillations	
	3100–2800	Aromatic C-H/CH_2_ stretching oscillations	
CC	1635	H-O-H bending oscillations (of crystal water)	[40]
BMIMCl	1166	C-N stretching oscillations	[41]
	1573	Imidazolidinium framework oscillations	

**Table 2:** The data on density (ρ), porosity (µ), total pore volume (V_p_), and water absorption coefficient (E) of pristine MFC/PS matrix and matrices impregnated with CC and BMIMCl.

Sample	ρ, g/cm^3^	V_p_, cm^3^	µ, %	E
MFC/PS	0.05	60.0 ± 1.3	29.4 ± 0.6	2.80
MFC/PS/CC	0.25	20.0 ± 2.3	9.8 ± 1.1	2.60
MFC/PS/BMIMCl	0.44	93.5 ± 4.2	45.8 ± 2.1	3.80

**Table 3:** Tensile strength (σ_max_), tearing strength (σ_p_), maximum tensile load (P_max_), and breaking load (**P_p_**) of pristine MFC/PS matrix and matrices impregnated with CC and BMIMCl.

Sample	σ_max_, MPa	σ_p_, MPa	P_max_, N	P_p_, N
MFC/PS	2.4 ± 0.4	1.7 ± 0.6	82.0 ± 1.7	55.2 ± 12.5
MFC/PS/CC	2.6 ± 0.2	1.8 ± 0.4	83.6 ± 1.4	53.5 ± 2.9
MFC/PS/BMIMCl	2.0 ± 0.1	1.5 ± 0.7	79.1 ± 1.1	39.8 ± 5.4

**Table 4:** Moisture sorption by composites at different air humidities.

**Absorption** **Conditions**	**Weight of Absorbed Moisture per Sample Weight, %**			
	**Time**	**MFC/PS**	**MFC/PS/BMIMCl**	**MFC/PS/CC**
T = 25 °C φ = 28%	150 min	1.6 *±* 0.3	3.4 *±* 0.1	1.4 *±* 0.2
	24 h	3.9 *±* 0.5	12.1 *±* 0.2	4.6 *±* 0.4
T = 25 °C φ = 63%	150 min	1.9 *±* 0.2	5.3 *±* 0.3	11.0 *±* 0.3
	24 h	5.9 *±* 0.4	16.7 *±* 0.5	16.9 *±* 0.5
**Desorption** **Conditions**	**Sample Weight Reduction per Absorbed Water Weight, %**			
	**Time**	**MFC/PS**	**MFC/PS/BMIMCl**	**MFC/PS/CC**
T = 55 °C φ = 8%	150 min	99.2 *±* 0.1	74.7 *±* 2.3	152.6 *±* 3.8
	24 h	99.8 *±* 0.3	94.8 *±* 2.4	184.8 *±* 4.3

## Data Availability

Data are available from the corresponding author by email upon reasonable request.

## References

[1] Zeng W., You T., Wu W. (2024). Passive Atmospheric Water Harvesting: Materials, Devices, and Perspectives. Nano Energy.

[2] Kandeal A.W., Joseph A., Elsharkawy M., Elkadeem M.R., Hamada M.A., Khalil A., Eid Moustapha M., Sharshir S.W. (2022). Research Progress on Recent Technologies of Water Harvesting from Atmospheric Air: A Detailed Review. Sustain. Energy Technol. Assess..

[3] Ahmad M., Nighojkar A., Plappally A. (2023). A Review of the Methods of Harvesting Atmospheric Moisture. Environ. Sci. Pollut. Res..

[4] Wang M., Liu E., Jin T., Zafar S., Mei X., Fauconnier M.-L., De Clerck C. (2024). Towards a Better Understanding of Atmospheric Water Harvesting (AWH) Technology. Water Res..

[5] Nikkhah H., Azmi W.M.B.W., Nikkhah A., Najafi A.M., Babaei M.M., Fen C.S., Nouri A., Mohammad A.W., Lun A.W., Yong N.L. (2023). A Comprehensive Review on Atmospheric Water Harvesting Technologies: From Thermodynamic Concepts to Mechanism and Process Development. J. Water Process Eng..

[6] Zhang S., Fu J., Xing G., Zhu W., Ben T. (2023). Porous Materials for Atmospheric Water Harvesting. ChemistryOpen.

[7] Lu W., Ong W.L., Ho G.W. (2023). Advances in Harvesting Water and Energy from Ubiquitous Atmospheric Moisture. J. Mater. Chem. A.

[8] Zhou X., Lu H., Zhao F., Yu G. (2020). Atmospheric Water Harvesting: A Review of Material and Structural Designs. ACS Mater. Lett..

[9] Pan T., Yang K., Han Y. (2020). Recent Progress of Atmospheric Water Harvesting Using Metal-Organic Frameworks. Chem. Res. Chin. Univ..

[10] Vikrant K., Kumar V., Kim K.-H., Kukkar D. (2017). Metal–Organic Frameworks (MOFs): Potential and Challenges for Capture and Abatement of Ammonia. J. Mater. Chem. A.

[11] Gutiérrez M., Zhang Y., Tan J.-C. (2022). Confinement of Luminescent Guests in Metal–Organic Frameworks: Understanding Pathways from Synthesis and Multimodal Characterization to Potential Applications of LG@MOF Systems. Chem. Rev..

[12] Li T., Yu H., Mi J., Li C., Meng H., Jin J. (2023). Highly Hydrophilic Acrylate Copolymer Supported MIL-160 for Air Water Harvesting. Chem. Phys. Lett..

[13] Feng A., Akther N., Duan X., Peng S., Onggowarsito C., Mao S., Fu Q., Kolev S.D. (2022). Recent Development of Atmospheric Water Harvesting Materials: A Review. ACS Mater. Au.

[14] Chen Y., Mu T. (2021). Revisiting Greenness of Ionic Liquids and Deep Eutectic Solvents. Green Chem. Eng..

[15] Lu H., Shi W., Guo Y., Guan W., Lei C., Yu G. (2022). Materials Engineering for Atmospheric Water Harvesting: Progress and Perspectives. Adv. Mater..

[16] Gong F., Li H., Zhou Q., Wang M., Wang W., Lv Y., Xiao R., Papavassiliou D.V. (2020). Agricultural Waste-Derived Moisture-Absorber for All-Weather Atmospheric Water Collection and Electricity Generation. Nano Energy.

[17] Farghal H.H., Nebsen M., El-Sayed M.M.H. (2023). Exploitation of Expired Cellulose Biopolymers as Hydrochars for Capturing Emerging Contaminants from Water. RSC Adv..

[18] Dong J., Chen X., Li Y., Luan M., Yang X., Chen H., Koosha M., Zhai Y., Fakhrullin R.F. (2024). Hydrophilic Chitosan: Modification Pathways and Biomedical Applications. Russ. Chem. Rev..

[19] Hong F., Qiu P., Wang Y., Ren P., Liu J., Zhao J., Gou D. (2024). Chitosan-Based Hydrogels: From Preparation to Applications, a Review. Food Chem. X.

[20] Yu F., Chen Z., Guo Z., Irshad M.S., Yu L., Qian J., Mei T., Wang X. (2020). Molybdenum Carbide/Carbon-Based Chitosan Hydrogel as an Effective Solar Water Evaporation Accelerator. ACS Sustain. Chem. Eng..

[21] Zainal S.H., Mohd N.H., Suhaili N., Anuar F.H., Lazim A.M., Othaman R. (2021). Preparation of Cellulose-Based Hydrogel: A Review. J. Mater. Res. Technol..

[22] Omidian H., Akhzarmehr A., Chowdhury S.D. (2024). Advancements in Cellulose-Based Superabsorbent Hydrogels: Sustainable Solutions across Industries. Gels.

[23] Wei P., Chen W., Song Q., Wu Y., Xu Y. (2021). Superabsorbent Hydrogels Enhanced by Quaternized Tunicate Cellulose Nanocrystals with Adjustable Strength and Swelling Ratio. Cellulose.

[24] Kallenberger P.A., Fröba M. (2018). Water Harvesting from Air with a Hygroscopic Salt in a Hydrogel–Derived Matrix. Commun. Chem..

[25] Li S., Hernandez S., Salazar N. (2023). Biopolymer-Based Hydrogels for Harvesting Water from Humid Air: A Review. Sustainability.

[26] Bai Q., Zhou W., Cui W., Qi Z. (2024). Research Progress on Hygroscopic Agents for Atmospheric Water Harvesting Systems. Materials.

[27] Bai Z., Wang P., Xu J., Wang R., Li T. (2024). Progress and Perspectives of Sorption-Based Atmospheric Water Harvesting for Sustainable Water Generation: Materials, Devices, and Systems. Sci. Bull..

[28] Ejeian M., Wang R.Z. (2021). Adsorption-Based Atmospheric Water Harvesting. Joule.

[29] Nguyen L.T., Bai Z., Zhu J., Gao C., Liu X., Wagaye B.T., Li J., Zhang B., Guo J. (2021). Three-Dimensional Multilayer Vertical Filament Meshes for Enhancing Efficiency in Fog Water Harvesting. ACS Omega.

[30] William G.E., Mohamed M.H., Fatouh M. (2015). Desiccant System for Water Production from Humid Air Using Solar Energy. Energy.

[31] Safoui R., Belaaribi R., Achahour O., Elfanaoui A., Ihlal A., Mouaky A., Amagour M.E.H., Abou Oualid H., Awad M.M. (2024). Atmospheric Water Harvesting Using a Desiccant-Based Solar Still: Experimental Investigation and Economic Analysis. Eng. Res. Express.

[32] Li J., Yao Z., Liu X., Yang C., Liu P., Liu P., Wang W., Guo H., Huang G., Jin X. (2025). Modified Metal-Organic Framework-Based Polymer Materials for Atmospheric Water Harvesting and Liquid Water Storage. Surf. Interfaces.

[33] Wang X., Yang D., Zhang M., Hu Q., Gao K., Zhou J., Yu Z.-Z. (2022). Super-Hygroscopic Calcium Chloride/Graphene Oxide/Poly(N-Isopropylacrylamide) Gels for Spontaneous Harvesting of Atmospheric Water and Solar-Driven Water Release. ACS Appl. Mater. Interfaces.

[34] Yang X., Chen Z., Xiang C., Shan H., Wang R. (2024). Enhanced Continuous Atmospheric Water Harvesting with Scalable Hygroscopic Gel Driven by Natural Sunlight and Wind. Nat. Commun..

[35] Kim H., Rao S.R., Kapustin E.A., Zhao L., Yang S., Yaghi O.M., Wang E.N. (2018). Adsorption-Based Atmospheric Water Harvesting Device for Arid Climates. Nat. Commun..

[36] Kim H., Yang S., Rao S.R., Narayanan S., Kapustin E.A., Furukawa H., Umans A.S., Yaghi O.M., Wang E.N. (2017). Water Harvesting from Air with Metal-Organic Frameworks Powered by Natural Sunlight. Science.

[37] Cherednichenko K., Bardina K., Vishnevich A., Gablina M., Gataulina A., Nikolaev Y., Gushchin P., Ivanov E., Kopitsyn D., Vinokurov V. (2023). A Facile One-Step Synthesis of Polystyrene/Cellulose (PS@MFC) Biocomposites for the Preparation of Hybrid Water-Absorbing Sponge Materials. Polymers.

[38] Cherednichenko K.A., Sayfutdinova A.R., Kraynov A., Anikushin B., Ignatiev V., Rubtsova M.I., Konstantinova S.A., Shchukin D.G., Vinokurov V.A. (2022). A Rapid Synthesis of Nanofibrillar Cellulose/Polystyrene Composite via Ultrasonic Treatment. Ultrason. Sonochem..

[39] Fang J., Xuan Y., Li Q. (2010). Preparation of Polystyrene Spheres in Different Particle Sizes and Assembly of the PS Colloidal Crystals. Sci. China Technol. Sci..

[40] Cao S., Luo X., Han X., Lu X., Zou C. (2022). Development of a New Modified CaCl_2_·6H_2_O Composite Phase Change Material. Energies.

[41] Chen K., Xu W., Ding Y., Xue P., Sheng P., Qiao H., Wang S., Yu Y. (2020). Mechanical and Thermal Properties of All-Wood Biocomposites through Controllable Dissolution of Cellulose with Ionic Liquid. Polymers.

[42] Li Y., Liu Q., Liu Y., Wang D., Song W., Chen Y., Liu J. (2020). Calcium Chloride Hexahydrate/Nano-SiO_2_ Composites as Form-Stable Phase Change Materials for Building Energy Conversation: The Influence of Pore Size of Nano-SiO_2_. Energy Build..

[43] Kadokawa J., Murakami M., Kaneko Y. (2008). A Facile Preparation of Gel Materials from a Solution of Cellulose in Ionic Liquid. Carbohydr. Res..

[44] Aghmih K., Bouftou A., El Bouchti M., Boukhriss A., Gmouh S., Majid S. (2023). Synthesis and Application of Functionalized Ionic Liquids-Based Imidazolium as Solvent for Cotton Fibre Cellulose Dissolution. Cellulose.

[45] LaPotin A., Zhong Y., Zhang L., Zhao L., Leroy A., Kim H., Rao S.R., Wang E.N. (2021). Dual-Stage Atmospheric Water Harvesting Device for Scalable Solar-Driven Water Production. Joule.

[46] Nasef M. (2004). Preparation and Applications of Ion Exchange Membranes by Radiation-Induced Graft Copolymerization of Polar Monomers onto Non-Polar Films. Progress Polym. Sci..

